# Long Non-Coding RNAs and RNA-Binding Proteins in Pancreatic Cancer Development and Progression

**DOI:** 10.3390/cancers17101601

**Published:** 2025-05-08

**Authors:** Pit Preckwinkel, Khursheed Ul Islam Mir, Florian W. Otto, Hend Elrewany, Andrea Sinz, Stefan Hüttelmaier, Nadine Bley, Tony Gutschner

**Affiliations:** 1 Section for RNA Biology and Pathogenesis, Institute of Molecular Medicine, Faculty of Medicine, Martin Luther University Halle-Wittenberg, 06120 Halle (Saale), Germany; pit.preckwinkel@uk-halle.de; 2 Section for Molecular Cell Biology, Institute of Molecular Medicine, Faculty of Medicine, Martin Luther University Halle-Wittenberg, 06120 Halle (Saale), Germany; khursheed.mir@medizin.uni-halle.de (K.U.I.M.); hend.elrewany@medizin.uni-halle.de (H.E.); stefan.huettelmaier@medizin.uni-halle.de (S.H.); 3 Department of Pharmaceutical Chemistry and Bioanalytics, Institute of Pharmacy, Faculty of Natural Sciences I, Martin Luther University Halle-Wittenberg, 06120 Halle (Saale), Germany; florian.otto@pharmazie.uni-halle.de (F.W.O.); andrea.sinz@pharmazie.uni-halle.de (A.S.); 4 Center for Structural Mass Spectrometry, Martin Luther University Halle-Wittenberg, 06120 Halle (Saale), Germany

**Keywords:** HuR, IGF2BP1, KRAS, LINC00261, LncRNA, MAPK, PDAC, RBP, TGF-β

## Abstract

Pancreatic ductal adenocarcinoma (PDAC) is the most common type of pancreatic cancer and is responsible for about 467,000 cancer deaths annually. An oftentimes asymptomatic early phase of this disease results in a delayed diagnosis, and patients often present with advanced disease. Current treatment options have limited survival benefits, and only a minor patient population carries actionable genomic alterations. Hence, innovative personalized treatment strategies that consider molecular, cellular and functional analyses are urgently needed for pancreatic cancer patients. However, the majority of the genetic alterations found in PDAC are currently undruggable, or patients’ response is not as expected. Therefore, non-genomic biomarkers and alternative molecular targets should be considered in order to advance the clinical management of PDAC patients. In line with this, recent gene expression and single-cell transcriptome analyses have identified molecular subtypes and transcriptional cell states that affect disease progression and drug efficiency. In this review, we will introduce long non-coding RNAs (lncRNAs) as well as RNA-binding proteins (RBPs) that are able to modulate the transcriptome of a cell through diverse mechanisms, thereby contributing to disease progression. We will provide a brief overview about the general functions of lncRNAs and RBPs, respectively. Subsequently, we will highlight selected lncRNAs and RBPs that have been shown to play a role in PDAC development, progression and drug response. Finally, we will present strategies aiming to interfere with the expression and function of lncRNAs and RBPs.

## Simple Summary

Pancreatic cancer is a deadly disease which is often detected at advanced stages. Novel and innovative approaches are urgently needed to improve diagnosis and personalized treatment strategies to achieve significant clinical benefits. Here, we introduce two interesting yet largely understudied classes of disease-associated molecules, i.e., long non-coding RNAs and RNA-binding proteins. We highlight their potential as biomarkers as well as therapeutic targets, and we provide examples of their functional diversity contributing to pancreatic cancer development and progression.

## 1. Introduction

The most recent global cancer statistics for the year 2022 estimate a total of almost 20 million new cancer cases and about 9.7 million cancer deaths [1]. With nearly 510,716 new cases and 467,000 deaths, pancreatic cancer ranks at position 12 and 6 in terms of incidence and mortality, respectively. Pancreatic ductal adenocarcinoma (PDAC), the most common type of pancreatic cancer, is a devastating disease with an overall 5-year survival rate of approximately 13% [2]. An oftentimes asymptomatic early phase leading to a delayed diagnosis of PDAC in patients presenting with advanced and metastatic disease, in conjunction with a high resistance to currently available therapies, is a critical factor responsible for this high mortality rate. Of note, surgery as well as chemotherapy and radiotherapy is the major treatment option, and despite advances in surgical and perioperative approaches, high recurrence rates (up to 79% after three years) after curative intended surgery are observed [3]. Importantly, somatic mutations in key cancer genes such as *KRAS*, *TP53*, *CDKN2A*, and *SMAD4* as well as a large number of genomic and epigenetic passenger alterations generate a remarkable inter- and intratumoural heterogeneity, which induces a considerable variation in responses to anticancer therapies [4]. Moreover, the performance status and comorbidities of the patient often dictate the choice of regimen and limit the application of non-specific and toxic chemotherapy, thereby affecting therapy resistance and tumour progression. Hence, for pancreatic cancer patients, personalized treatment strategies that consider molecular, cellular and functional analyses are urgently needed to overcome these limitations. However, only 12–25% of pancreatic cancer cases harbour actionable genomic alterations [5]. For example, about 7% of patients carry *BRCA1/2* germline mutations that sensitize their tumours to the PARP inhibitor olaparib, which was shown to extend progression-free survival in metastatic pancreatic adenocarcinoma [6]. Additional targeted therapies for rare molecular pancreatic cancer subgroups like *NTRK*-fusion-positive, the *KRAS^G12C^* mutant, DNA mismatch repair-deficient, or microsatellite-instable (MSI^high^) tumours might be viable options as well [7,8,9,10]. However, the majority of the genetic alterations found in PDAC are currently undruggable, or patients’ response is not as expected despite targeting actionable mutations with tailored drugs. Therefore, non-genomic biomarkers and alternative molecular targets should be considered in order to advance the clinical management of PDAC patients. In line with this, recent gene expression and single-cell transcriptome analyses have identified molecular subtypes and transcriptional cell states that affect disease progression and drug efficiency [11,12,13,14].

In this review, we will introduce long non-coding RNAs (lncRNAs) as well as RNA-binding proteins (RBPs), which have been shown to be associated with certain PDAC subtypes and are able to modulate the transcriptome of a cell through diverse mechanisms [15,16,17,18]. We will provide a brief overview about the general functions of lncRNAs and RBPs, respectively. Subsequently, we will highlight selected lncRNAs and RBPs that have been shown to play a role in PDAC development, progression, and drug response. Last but not least, we will present strategies aiming to interfere with the expression and function of lncRNAs and RBPs.

## 2. Role of lncRNAs in PDAC Development and Progression

Even though less than 2% of the human genome consists of protein-coding genes, transcription is pervasive, and modern RNA sequencing technologies have shown that transcripts are generated from 75 to 85% of the genome [19,20]. Some of these non-protein-coding RNAs (ncRNAs), such as ribosomal RNAs, transfer RNAs and microRNAs (miRNAs), can be categorized into classes based on their specific functions. What remains is a vast number of mostly RNA polymerase II (Pol II)-derived transcripts that vary in size, function and distribution. LncRNAs are somewhat arbitrarily, as they are defined as transcripts that are longer than 500 nucleotides (nts) [21]. More than 100,000 human lncRNAs have been recorded, exceeding the ~20,000 protein-coding genes in number. Their lengths range from one to tens of thousands of nucleotides, and they can occupy various genomic locations [22,23,24]. Intergenic lncRNAs are derived from regions devoid of coding potential, while intronic and sense/antisense lncRNAs are co-localized with protein-coding genes. Bidirectional lncRNAs share promoters with protein-coding genes and are transcribed in the antisense direction. Many lncRNAs share features with messenger RNAs (mRNAs) in that they are often polyadenylated and 5′capped, tend to comprise multiple exons and undergo alternative splicing [25,26]. Major differences lie in their overall lower degree of sequence conservation between species and their more restricted expression patterns, often being limited to specific cell types or developmental stages [27,28].

On a molecular level, lncRNAs can form complex secondary and tertiary structures that can interface with DNA, proteins and other RNAs and may be found in both nucleus and cytoplasm, enabling them to regulate gene expression on every level [29]. Through RNA-DNA interactions, they can alter the chromatin architecture and control transcription [30,31]. Via RNA-RNA actions, they affect the processing, splicing, editing, stability and localization of RNAs, in general, as well as influence the translation rates of mRNAs [32,33,34,35]. Finally, they can regulate protein localization, turnover and modification through RNA–protein interactions and serve as scaffolds for large protein complexes and biomolecular condensates [36,37,38,39,40]. When it comes to classifying lncRNAs based on their functions, the broadest approach is to differentiate between cis-acting and trans-acting lncRNAs. Cis-acting lncRNAs accumulate at and interact with the locus from which they are transcribed [41]. Many lncRNAs are found associated with the chromatin, tethered to their transcriptional origins by Pol II, and these proximity-based mechanisms may offset their often low expression levels [42,43]. Other lncRNAs vacate their sites of transcription to operate in trans and exert their functions in other nuclear regions or the cytoplasm. Table 1 lists a selection of common modes of action that have been found for lncRNAs acting in both cis and trans. Notably, a given lncRNA can have multiple interaction partners and functional modes depending on the biological context.

Importantly, lncRNAs have been associated with a broad spectrum of human diseases, and it was discovered that the vast majority (~99%) of somatic single-nucleotide variations (SNVs) occur in non-coding regions including lncRNA loci [62]. Importantly, gene expression analyses uncovered a frequent dysregulation of lncRNAs in cancer, and functional studies identified individual non-coding transcripts controlling cancer hallmarks [63,64,65]. Accordingly, lncRNAs have gained relevance as biomarkers for the early detection of cancer, prognostic markers and therapeutic targets [66]. Their expression patterns also have the tendency to be more cancer-type specific compared to pseudogenes and protein-coding genes, further underscoring their diagnostic value [67]. Given the dire trajectory of pancreatic cancer incidence and mortality, the potential for new biomarkers and therapeutic avenues offered by lncRNAs holds great potential. Of note, a large selection of lncRNAs has been found to be dysregulated in pancreatic tumours and PDAC cell lines and was shown to be associated with clinical disease parameters [68,69,70,71,72]. Many of these transcripts are upregulated in PDAC acting as oncogenes, promoting proliferation, survival and invasiveness. Several lncRNAs that were previously known to promote tumours in other cancer types have also been found to be increased in PDAC, e.g., H19, *MALAT1* and *HOTAIR* [73,74,75]. Tumour-suppressive lncRNAs have also been identified, with examples including *DGCR5*, *MEG3* and *lnc-PCTST* [76,77,78]. In the following paragraphs, we will highlight individual lncRNAs that have been linked with PDAC development and oncogenic signalling pathways underlying pancreatic cancer progression.

### 2.1. LncRNAs Associated with PDAC Initiation and Early Development

Frequently, dysregulated lncRNAs in PDAC are identified by comparing the gene expression signatures of late-stage tumour samples and normal pancreatic tissues. In order to gain insights into the early stages of carcinogenesis, precursor lesions or preceding benign pancreatic diseases should be included in such analyses, and liquid biopsy samples might be considered for early biomarker discovery. In fact, the lncRNAs *ABHD11-AS1* and *HULC* were both detected in the serum of pancreatic cancer patients with increasing levels following the disease’s progression from healthy patients to those with benign diseases like chronic pancreatitis, as well as to those with fully developed pancreatic cancer [79,80]. Another study focused on intraductal papillary mucinous neoplasms (IPMNs), a common type of cystic PDAC precursor lesion. LncRNA signatures in the plasma from a cohort of IPMN cases could differentiate both IPMNs from non-disease controls as well as aggressive IPMNs from benign precursors [81]. However, patient biopsies from early-stage PDAC cases are often difficult to obtain due to the late diagnosis of the disease. Here, genetically engineered mouse models (GEMMs) present a powerful tool to follow the entire progression, from precursor to metastatic cancer, and study the role of lncRNAs in this cascade. For example, Mello et al. used a mouse model with pancreas-specific expression of oncogenic *Kras^G12D^* to study the role of the lncRNA *Neat1* in tumour initiation. In this model, *Neat1* proved critical in suppressing the development of premalignant pancreatic intraepithelial neoplasia (PanIN) and cystic lesions [82]. Of note, this conserved lncRNA has been implicated in a variety of cellular functions and is best known for its architectural role in nuclear paraspeckles, a type of nuclear body which is itself multifunctional [83]. It is therefore perhaps not surprising that its role in cancer is also multifaceted, promoting or suppressing tumour development depending on the cancer type and context. Indeed, in developed pancreatic cancer and fully transformed PDAC cell lines, human *NEAT1* has mostly been assigned oncogenic roles [84,85,86]. The example of *NEAT1* highlights the importance of resolving the functions of lncRNAs in early cancer stages to gain a complete mechanistic understanding of the disease and the stage-specific contributions of lncRNAs.

### 2.2. LncRNAs Associated with Oncogenic KRAS Signalling

In the majority of PDAC cases, mutations cause a molecular switch in KRAS to assume a constitutive ‘ON’ state, resulting in a constant stream of proliferation and survival signals. The G12D and G12V mutations in the *KRAS* gene are sufficient to initiate the development of precursor lesions, and the gene dosage of mutant *KRAS* remains relevant throughout the full progression of the disease up to the growth and maintenance of metastases [87,88,89]. Interestingly, some lncRNAs have been found to regulate the expression and activity of *KRAS*. For example, *MALAT1* and *NUTF2P3-01* were both found to be overexpressed in pancreatic cancer, and their knockdown led to a reduction in KRAS protein level. Both lncRNAs share miRNA binding sites with the *KRAS* mRNA, suggesting a ceRNA network that is involved in tuning intracellular *KRAS* expression [90,91]. Additionally, several studies have reported the upregulation of the lncRNA *UCA1* in PDAC tissues and cell lines [92]. One of these studies observed that *UCA1* promoted the interaction between the KRAS and hnRNPAB1 proteins [93]. This interaction had previously been shown to positively regulate the activation of PI3K/AKT/mTOR signalling through oncogenic KRAS [94]. The authors also observed that *UCA1* knockdown decreased KRAS expression, and KRAS depletion reduced *UCA1* levels, indicating a positive feedback loop [93]. Furthermore, *UCA1* was found to modulate the downstream signalling pathways of KRAS. In particular, MAPK/ERK signalling was shown to be positively regulated by *UCA1*, which increased mitochondrial dynamics and thereby affected the migratory ability of pancreatic cancer cells [95]. Another study corroborated the enhancement of ERK signalling by *UCA1* and revealed that *CUDR*, an alternative transcript variant of *UCA1*, could promote the migration and invasion of pancreatic cancer cells by activating AKT/FAK and ERK signalling to induce an epithelial-to-mesenchymal transition (EMT) [96,97].

In addition to *UCA1*, several other lncRNAs have been shown to modulate KRAS downstream signalling in pancreatic cancer. For example, *ABHD11-AS1* and *SNHG1* are both overexpressed in PDAC and promote the proliferation and survival of cancer cells by up-regulating PI3K/AKT signalling [98,99]. In addition, the lncRNA *LUCAT1* was found to increase both AKT- and p38-mediated MAPK signalling, thereby supporting proliferation and invasion [100,101]. One lncRNA that has been studied in more detail on the mechanistic level is *LINC01232*. Like *UCA1*, it has also been shown to interact with hnRNPAB1 [102]. In addition to the *KRAS* mRNA, hnRNPAB1 also binds to the pre-mRNA of the downstream effector *ARAF* and regulates its splicing. This interaction favours the generation of the full-length, kinase-proficient ARAF protein [103]. *LINC01232* stabilizes hnRNPAB1 by reducing its ubiquitination and proteasomal degradation, thereby promoting the splicing of the active ARAF isoform and subsequent downstream MAPK signalling [102].

In addition to PDAC-associated lncRNAs that regulate KRAS and its downstream pathways, several studies have also identified non-coding transcripts whose expression is regulated by these signalling cues. For instance, a recent study systematically deactivated MAPK/ERK signalling in a panel of PDAC cell lines and performed comparative transcriptome analyses to identify MAPK-associated lncRNAs [104]. Out of a list of 45 candidates, *LINC00941* showed the most consistent downregulation upon MAPK abrogation, and the authors identified a binding site for the MAPK-activated transcription factor ETS-1 in its promoter region [104]. *LINC00941* has been linked to PDAC progression in several studies and has been implicated in enhancing aerobic glycolysis by modulating the Hippo pathway and inducing AKT/FAK signalling by binding and stabilizing the ANXA2 protein [105,106]. Overall, these findings highlight the intimate link between lncRNAs and KRAS/MAPK/ERK and their associated downstream signalling in PDAC.

### 2.3. LncRNAs Associated with Oncogenic and Tumour-Suppressive TGF-β Signalling

TGF-β signalling has an ambivalent role throughout PDAC carcinogenesis. In the early stages of the disease, it presents a serious impediment to cancer progression by blocking the cell cycle and inducing apoptosis. In contrast, advanced tumours benefit from TGF-β-mediated immune evasion and the increased cell motility and metastatic potential stemming from a TGF-β-mediated epigenetic and transcriptional reprogramming that leads to EMT [107]. The loss of the TGF-β downstream signalling protein and tumour suppressor SMAD4, which occurs in up to 60% of PDAC, is often instrumental in this switch [108,109,110,111]. Many of the tumour suppressive effects of TGF-β are mediated by SMAD4, while the oncogenic pathways are mostly SMAD4-independent [112]. Intriguingly, recent studies identified regulatory lncRNAs that modulate the expression of SMAD4 in pancreatic cancer cells. One prominent lncRNA that has been extensively studied in cancer, in part due to the close proximity of its genomic locus to the well-known *MYC* oncogene, is *PVT1* [113,114]. Increased levels of *PVT1* were detected in PDAC tissues and cell lines, and its depletion inhibited TGF-β/SMAD signalling, including the phosphorylation of SMAD2 and SMAD3, as well as the expression of TGF-β1, but at the same time, it increased SMAD4 levels. Conversely, transient overexpression of *PVT1* decreased SMAD4 levels, enhanced cellular proliferation and activated an EMT programme, thereby promoting cell migration and invasion [115]. In addition, *LINC00909* was recently identified as an upregulated lncRNA in PDAC tissues compared to a normal pancreas, and its expression was associated with poor clinicopathological features and patient outcomes. Importantly, experimental studies revealed a cytoplasmic localization of this lncRNA in PDAC cell lines, and the modulation of its expression inversely affected the stability of the *SMAD4* mRNA. Hence, this study established *LINC00909* as an important regulator of pluripotency factors, cancer stemness and metastasis by inhibiting *SMAD4* expression [116].

While the downregulation or inactivation of *SMAD4* is important for PDAC progression, the TGF-β pathway has several other members whose expression or activity could be modulated by lncRNAs. In fact, several studies have identified lncRNAs that apply diverse modes of action in order to promote or inhibit TGF-β signalling in PDAC. Two lncRNAs, namely *BC037916* and *LINC00462*, were found to be upregulated in PDAC, and they positively regulate the core TGF-β pathway, as evaluated by the increased phosphorylation of SMAD2/3 [117,118]. *BC037916* was shown to activate JAK/STAT in addition to SMAD signalling, thereby promoting an EMT programme that increased cellular invasion and metastasis in xenograft models [117]. In contrast, *LINC00462* was found to sequester miR-665, thereby upregulating TGFBR1 and TGFBR2 protein levels and activating migration and invasion through EMT induction [118].

In addition to these oncogenic lncRNAs, previous studies also highlighted the role of lncRNAs acting as tumour suppressors by modulating TGF-β signalling. For example, *LINC-PINT* was found to be downregulated in PDAC, and low plasma *LINC-PINT* expression might serve as a biomarker for early pancreatic cancer detection, whereas low levels of *LINC-PINT* in tumour tissues correlated with a poor prognosis after pancreatectomy [119,120]. Of note, the plasma levels of *LINC-PINT* and TGF-β1 were positively correlated in early-stage PDAC patients but not in healthy controls. Furthermore, the overexpression of *LINC-PINT* upregulated TGF-β1 expression in a pancreatic cancer cell line but not in telomerase-immortalized pancreatic ductal cells [120]. Importantly, *LINC-PINT* overexpression as well as exogenous TGF-β1 stimulation specifically reduced the proliferation of the pancreatic cancer cell line without affecting the growth of the non-malignant immortalized cells. The inhibitory effects of both *LINC-PINT* and TGF-β1 could be blocked by the simultaneous application of SD-208, a TGFBR1 inhibitor [120]. In conclusion, *LINC-PINT* may inhibit early-stage PDAC growth through TGF-β pathway activation. Last but not least, our own studies have identified *LINC00261* as a TGF-β-regulated tumour-suppressive lncRNA that is involved in maintaining a pro-epithelial cell state, which is associated with a favourable disease outcome. We showed that the depletion of *LINC00261* induced the expression of EMT-related genes, decreased E-cadherin levels and increased cell migration and invasion [121].

**Figure 1:**
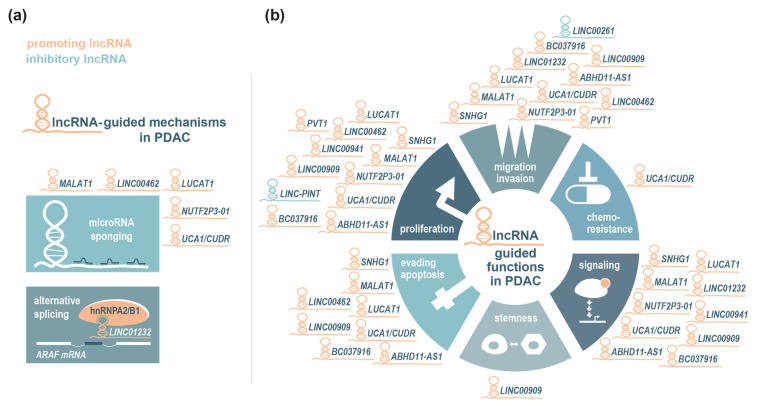
Long non-coding RNA (lncRNA)-guided functions and regulatory mechanisms involved in PDAC development and progression. Identified mechanisms (**a**) and cellular functions (**b**) of selected lncRNAs in pancreatic cancer, specifically in the context of KRAS/MAPK and TGF-/SMAD signalling, have been described in the main text and are summarized here schematically, with implicated promoting (orange) or inhibitory (turquois) lncRNAs assigned to their respective function. For more details about the clinical relevance and the mode of action of these lncRNAs, see Table 2.

These examples underscore the diagnostic potential and functional relevance of lncRNAs in modulating the oncogenic as well as tumour-suppressive properties of the TGF-β signalling pathway in pancreatic cancer cells. Table 2 lists and Figure 1 schematically summarizes the findings on lncRNAs associated with KRAS and TGF-β signalling that have been described in this section. These principal cellular pathways controlling early PDAC progression evidently undergo regulation by lncRNAs on many levels.

The large number and functional versatility of lncRNAs, in general, suggest that this list is by no means completed yet. Systematic approaches towards mapping lncRNA dependencies could reveal many more potential biomarkers and therapeutic targets. Future work should incorporate samples from patients with benign precursor lesions and early-stage cancer models whenever possible. As illustrated by the examples of *NEAT1* and the TGF-β pathway, the roles of lncRNAs and the processes involving them may change dynamically as pancreatic cancer progresses.

## 3. Role of RNA-Binding Proteins in PDAC

**Figure 2:**
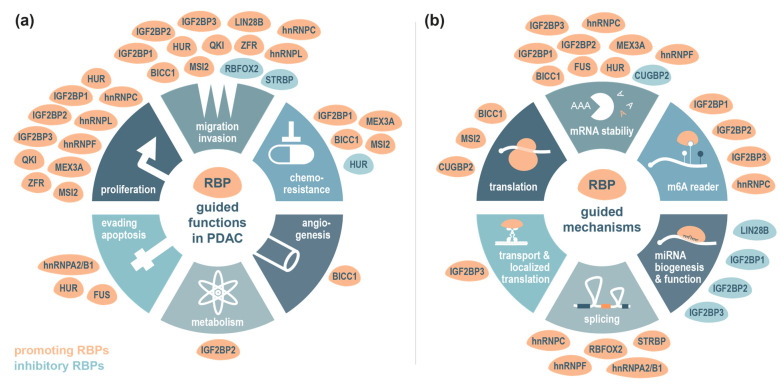
RNA-binding protein-guided functions and regulatory mechanisms involved in PDAC development and progression. Indicated cellular (**a**) and molecular functions (**b**) are depicted schematically with implicated promoting (orange) or inhibitory (turquois) RBPs assigned to their respective functions. Importantly, several RBPs have been shown to modulate cancer hallmarks by applying one or more modes of action to regulate their target transcripts.

RNA-binding proteins are essential regulators of RNA biology, overseeing key stages of RNA life, including RNA splicing, processing, transport, stability, turnover and translation [123,124,125]. These multifunctional proteins are integral for maintaining cellular homeostasis and, consequently, human health. However, RBPs can become dysregulated, contributing to various diseases including cancer [126]. In PDAC, several RBPs are considered as disease drivers with prognostic relevance [127]. Some RBPs like LIN28A/B, ELAVL1, IGF2BP or MUSASHI proteins also exhibit long-standing and well-characterized roles in carcinogenesis or tumour suppression in other cancer types. In these cases, small-molecule inhibitors have been developed and employed to target malignant phenotypes. Despite these advancements, novel cancer-related functions for RBPs have recently been identified, continuously increasing the portfolio of RBP-based therapeutic strategies. This is particularly relevant in the context of PDAC where emerging evidence links specific RBPs to disease progression. In this section, we explore recent pancreatic cancer-related findings of selected RBPs, delving into the molecular mechanisms that underpin their influence on tumour growth, survival and metastasis (Figure 2). We will provide an overview of the regulatory pathways affected by the selected RBPs and summarize the potential of targeting these proteins.

### 3.1. ELAV-Like RNA Binding Protein 1 (ELAVL1)

ELAVL1, also known as human antigen R (HuR), plays a crucial role in the post-transcriptional regulation of numerous mRNAs involved in proliferation, cell survival, the stress response and chemoresistance. The protein is primarily located in the nucleus but shuttles to the cytoplasm to control the expression of its target transcripts. HuR binds to AU- or U-rich elements in the 3′ untranslated regions (3′UTRs) of target mRNAs to stabilize and/or enhance the translation of the respective transcripts [128]. HuR was initially discovered to be overexpressed in various cancer entities associated with a poor prognosis [129,130]. In PDAC, the HuR protein was detected in the nucleus and cytoplasm, and its cytoplasmic localization was associated with the tumour (T) stage. Interestingly, high cytoplasmic expression of HuR was associated with a favourable outcome in patients who received adjuvant therapy with gemcitabine [131]. Mechanistically, it was shown that HuR promotes the expression of DCK, an enzyme that activates gemcitabine, by stabilizing the *DCK* mRNA and enhancing its translation [132]. However, a retrospective study conducted in a cohort of 175 patients with resected periampullary adenocarcinomas, including pancreatic cancer, could not confirm the association between HuR and DCK expression and found that high HuR levels or a high HuR cytoplasmic-to-nuclear ratio is associated with poor survival in patients that received gemcitabine [133]. This finding is well in line with other reports that have assigned tumour-promoting functions to HuR. For instance, HuR was shown to stabilize the *GPRC5A* mRNA, encoding an orphan G-protein-coupled receptor, which is upregulated by gemcitabine and contributes to resistance mechanisms in pancreatic cancer cells [134].

Furthermore, HuR was identified as a regulator of proliferation, the cell cycle and apoptotic pathways in PDAC-derived cells under normal and stress conditions. In detail, the treatment of pancreatic cancer cells with DNA-damaging agents led to an accumulation of HuR in the cytoplasm and silenced expression-sensitized cells to these agents. Mechanistically, HuR was identified as a post-transcriptional regulator of the mitotic inhibitor kinase WEE1 by stabilizing the corresponding mRNA of this kinase, especially after DNA damage. Importantly, the upregulation of WEE1 induces a G2/M cell cycle arrest that enables cancer cells to repair their DNA [135]. Another study confirmed this DNA repair-promoting function of HuR by regulating the expression of *BARD1* mRNA, thereby ensuring effective DNA repair via the homologous recombination repair pathway [136].

In addition to these DNA damage response-related functions, HuR was also shown to enhance the resilience of PDAC cells to cope with metabolic stress. For example, the depletion of HuR-sensitized pancreatic cancer cells to glucose deprivation led to increased apoptosis and reduced anchorage-independent colony formation. Interestingly, glucose deprivation induced the cytoplasmic translocation of HuR and allowed for the binding of this RBP to mRNAs encoding for several metabolic enzymes, including GPI, PRPS2 and IDH1. Of note, the depletion of HuR reduced the transcript and protein abundance of these metabolic enzymes in three pancreatic cancer cell lines, suggesting a critical role of HuR in modulating pancreatic cancer cell metabolism and survival under acute glucose deprivation [137]. This pro-survival effect of HuR in pancreatic cancer cells was observed in several other studies, and additional HuR target genes like *YAP1* and *DR5*/*TNFRSF10B* as well as their associated pathways have been shown to be activated or inhibited by HuR [138,139].

Besides proliferation and apoptosis, HuR has been shown to promote cancer cell migration, invasion and metastasis as well as stem cell properties through its regulation of key genes, such as *SNAI1*, that play a role in EMT [140]. Intriguingly, the interruption of the HuR-RNA interaction by a novel compound called KH-3 reduced PDAC cell viability, EMT and metastatic potential both in vitro and in vivo [140].

In addition to the aforementioned tumour cell intrinsic functions, HuR was also shown to contribute to the remodelling of the tumour microenvironment (TME) by regulating the secretion of PDGFA, which led to increased collagen deposition and stromal activation in PDAC [141].

In summary, given its multifaceted role in promoting PDAC cell growth and survival, drug resistance and DNA repair, metabolic adaptation, EMT as well as metastasis, HuR represents an interesting as well as challenging therapeutic target. Inhibiting HuR alone or in combination with, e.g., DNA-damaging agents or WEE1 inhibitors, may alter therapy responses and patient outcomes in pancreatic cancer.

### 3.2. Insulin-Like Growth Factor II mRNA-Binding Proteins (IGF2BPs)

The family of oncofetal IGF2BPs, commonly upregulated or de novo expressed in various solid cancers, is notably active in several types of cancer, including PDAC, where these RBPs regulate a range of cellular processes such as proliferation, migration, invasion, metabolic pathways, chemoresistance and interactions within the TME [142,143]. As highly conserved regulators of mRNA turnover and translation, IGF2BPs can act as N6-methyladenosine (m6A) readers, binding predominantly to the 3′UTR of target mRNAs [144,145]. Through this binding, IGF2BPs protect mRNAs from miRNA-mediated decay, thereby influencing the stability and translation efficiency of various oncogenic transcripts [146]. In detail, IGF2BP1 has been identified as a key player in pancreatic cancer and was found to be significantly upregulated in this malignancy [147,148]. The overexpression of IGF2BP1 was correlated with tumour size and shorter overall survival (OS) in pancreatic cancer patients, highlighting its clinical relevance as a prognostic marker [147,148]. Mechanistically, IGF2BP1 was shown to promote cell cycle progression and proliferation via CDC25A and E2F1 and by activating AKT signalling [147,148,149,150]. Moreover, IGF2BP1 was shown to contribute to gemcitabine resistance of pancreatic cancer cells via multiple mechanisms. One study unravelled a complex RNA regulatory network that is controlled by the interaction of IGF2BP1 with m6A-modified SH3BP5-AS1, which increased the expression and stability of this lncRNA. In turn, SH3BP5-AS1 activated the WNT signalling pathway by sponging miR-139-5p, upregulated CTBP1 expression and thereby contributed to increased pancreatic cancer cell invasion, migration and stemness, as well as enhanced resistance to gemcitabine [151]. Another study could show that gemcitabine treatment inhibited pancreatic cancer cell proliferation and migration and decreased the overall m6A level potentially via the treatment-induced downregulation of the m6A writer WTAP [152]. The reduction in WTAP led to a downregulation of the m6A-modified *MYC* mRNA as well as the MYC protein. Since the *MYC* mRNA is known to be bound and stabilized by IGF2BP1 in a m6A-dependent manner, the authors speculated that gemcitabine might interfere with the WTAP/MYC/IGF2BP1 axis to inhibit pancreatic cancer progression [152,153].

The second member of the IGF2BP family, namely IGF2BP2, was found to be overexpressed in pancreatic cancer tissues as well, where it has been associated with significantly shorter OS [154,155]. The overexpression of IGF2BP2 might be driven by multiple mechanisms, including the loss of tumour-suppressive miRNAs, gene amplification and upregulation mediated by the lncRNA *LINC00901* [154,156]. Interestingly, high IGF2BP2 expression is often found in high-risk tumours that exhibit lower immune response activity [157]. Moreover, a negative correlation with immune signature markers and a direct association with the expression of PD-L1, a key immune checkpoint protein, have been described for IGF2BP2. In fact, by regulating PD-L1 expression, IGF2BP2 may contribute to immune evasion, further promoting tumour survival in the immune-suppressive microenvironment of pancreatic cancer [158]. In addition, IGF2BP2 was shown to promote cell proliferation and metabolic reprogramming to support rapid tumour growth through the activation of the PI3K/AKT signalling pathway as well as through the stimulation of the glycolytic activity in PDAC cells by stabilizing the mRNA of *GLUT1*/*SLC2A1*, thus enhancing its expression level and facilitating increased glucose uptake [154,159].

Last but not least, the third family member, IGF2BP3, is most frequently used as a biomarker for solid tumours [160]. In 1997, IGF2BP3 was discovered in a large-scale screen for differentially expressed genes in pancreatic cancer, and it was cloned and initially named as a KH domain-containing protein overexpressed in cancer (KOC) [161]. Later studies confirmed its overexpression and revealed the prognostic relevance of IGF2BP3 in PDAC [161,162,163,164,165]. Importantly, based on biopsies or microdissections, IGF2BP3 is considered as a biomarker that detects early pancreatic lesions with high sensitivity and specificity [162,163,164,165,166,167,168]. Similar to IGF2BP2, IGF2BP3 is part of a prognostic five-gene signature that allows for the stratification of patients into low-risk and high-risk groups. The latter shows a high tumour mutational burden with lower response rates to immune checkpoint therapies [157]. Furthermore, elevated IGF2BP3 levels are associated with increased cell proliferation, migration and invasion, although its exact mechanisms remain incompletely understood. A recent study suggested that IGF2BP3 might act by blocking or mediating the interaction of specific transcripts with the RNA-induced silencing complex (RISC), thereby modulating their susceptibility to miRNA-mediated post-transcriptional gene silencing [169]. Further data suggest that IGF2BP3 might facilitate pancreatic cancer cell invasion and metastasis through additional mechanisms, including the regulation of localized translation at cell protrusions, altered adhesion by controlling CD44 expression or by stabilizing the *SMS* mRNA [170,171,172,173]. Furthermore, IGF2BP3 was shown to upregulate UBE2K, which reinforces the proliferation and stem cell-like phenotype in PDAC [174].

Collectively, these studies highlight the functional relevance of the individual IGF2BP family members in pancreatic cancer by employing overlapping as well as specific regulatory mechanisms to activate oncogenic pathways while at the same time inhibiting tumour suppressive genes and immune cell functions. Hence, these proteins are prime candidates for drug development efforts, and their oncofetal expression patterns should allow for cancer-specific targeting with minimal side effects.

### 3.3. BicC Family RNA-Binding Protein 1 (BICC1)

BICC1 plays a crucial role in regulating cell fate through post-transcriptional mechanisms [175]. Originally identified in *Drosophila melanogaster*, *BICC1* was recognized as a gene associated with maternal-effect mutations that cause a double-abdomen phenotype [176,177]. Later it was shown that the RBP BICC1 regulates anterior–posterior patterning in early development by controlling the localized translation of *oskar*, a key mRNA in Drosophila axis formation [178]. Recent studies highlight the upregulation of BICC1 in PDAC, particularly in lymph node metastases. Elevated BICC1 expression might affect immune cell infiltration and was shown to correlate with oncogenic pathways like EMT, TNFα/NF-kB and TGF-β signalling, which might underlie the poor prognosis of pancreatic cancer patients that express high levels of BICC1 in their tumours [179]. Mechanistically, BICC1 is able to influences tumour growth and progression through its post-transcriptional control of several molecular targets and pathways. In pancreatic cancer, BICC1 was shown to facilitate angiogenesis in a VEGF-independent manner [180]. In detail, an AU-rich sequence within the 3′UTR of the *LCN2* mRNA was recognized by BICC1, and this stabilizing interaction increased the expression of the LCN2 protein. Elevated levels of LCN2 subsequently activated the JAK2/STAT3 signalling pathway and resulted in the enhanced expression and secretion of the pro-angiogenic factor CXCL1. Intriguingly, the anti-tumour effect of gemcitabine could be improved by treating patient-derived xenograft models with elevated BICC1 expression with an anti-LCN2 antibody but not with the anti-VEGF antibody Bevacizumab [180]. Another possibility to improve gemcitabine efficacy in PDAC by targeting BICC1 and its downstream signalling was recently described. In particular, BICC1 has been identified as an activator of tryptophan catabolism by increasing the expression of IDO1 [181]. The resulting increase in tryptophan metabolites contributes to the synthesis of nicotinamide adenine dinucleotide (NAD+) and promotes oxidative phosphorylation, creating a metabolic environment conducive to a stem cell-like phenotype in cancer cells. Notably, the inhibition of the BICC1/IDO1/tryptophan metabolic axis has shown potential in improving the efficacy of gemcitabine by reducing drug resistance and limiting the stem cell-like qualities of tumour cells [181]. Hence, BICC1 emerges as a multifaceted regulator in pancreatic cancer, influencing processes such as EMT, immune infiltration, angiogenesis, chemoresistance and stemness. Its regulatory impact on *LCN2* and *IDO1* expression highlights its potential as a therapeutic target to overcome VEGF inhibitor resistance and improve responses to chemotherapy in PDAC.

Taken together, the RBPs introduced in this section highlight the important contribution of post-transcriptional regulators in modulating gene expression programmes in pancreatic cancers. Table 3 provides a comprehensive overview about additional pancreatic cancer-associated RBPs. While these RBPs represent interesting targets to treat PDAC, only a limited number of inhibitors targeting a specific RBP have been developed so far. In the final section, we will provide a general overview about the diverse opportunities that enable interference with RBPs as well as lncRNAs.

## 4. Strategies to Target lncRNAs and RBPs

**Figure 3:**
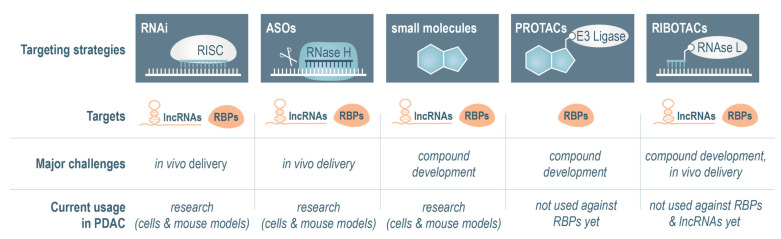
Summary of RBP- and lncRNA-directed therapeutic strategies and their current application in pancreatic cancer. Most strategies are currently at the early stages and have been tested in cell lines and pre-clinical mouse models. The targeting strategies can be divided into two groups. RNA interference (RNAi) and antisense oligonucleotides (ASOs). Ribonuclease-targeting chimeras (RIBOTACs) are generally RNA-directed approaches and can be used to degrade RBP-encoding mRNAs as well as lncRNAs. In contrast, small molecules and proteolysis-targeting chimeras (PROTACs) aim to target the RNA-binding protein directly and can be used to either interfere with its molecular interactions, i.e., binding to RNA, or to recruit an E3 ubiquitin ligase to induce the destruction of the RBP via the ubiquitin–proteasome system. Small molecules can also be directed against structural elements in lncRNAs to interfere with molecular interactions. The identification of specific and high-affinity binding compounds and the tumour-specific delivery of these substances pose major challenges for their clinical application in PDAC patients.

In general, targeting lncRNAs or RBPs in human PDAC patients is not a trivial task, and commonly applied strategies try to modulate the expression or interfere with molecular interactions. More recently, small molecules that mediate the selective degradation of RNAs and proteins have been developed and represent an innovative and promising opportunity (Figure 3). In this section, we will briefly introduce these strategies in more detail, and we discuss their advantages as well as their limitations.

### 4.1. RNA Interference-Based Targeting Approaches

RNA interference (RNAi) is a conserved cellular mechanism that utilizes small, double-stranded RNA molecules, such as small interfering RNA (siRNA), short hairpin RNA (shRNA) or miRNAs, to silence gene expression by targeting complementary RNA sequences [206]. This mechanism provides a versatile and fairly specific method for reducing lncRNA and RBP expression by effectively eliminating the respective transcripts that are mainly found in the cytoplasm. The specificity of the method minimizes the risk of off-target effects, which can be a concern with other therapeutic approaches. Furthermore, RNAi-based therapies offer the flexibility to inhibit multiple targets simultaneously, enabling a more comprehensive treatment design tailored to the complexities of the disease. Hence, this multifaceted approach can potentially address various pathways that contribute to tumour growth and resistance, enhancing overall treatment efficacy. However, despite its promising capabilities, RNA-based therapy faces significant challenges, particularly concerning delivery mechanisms. RNAi agents, such as siRNA and shRNA, are highly susceptible to rapid degradation in the bloodstream, which can significantly diminish their therapeutic potential. To achieve the effective silencing of target genes within PDAC cells, RNAi agents often require the use of specialized delivery vectors, such as lipid nanoparticles or viral vectors. These vectors play a crucial role in enhancing the stability of RNAi agents and facilitating their successful delivery to target cells. Consequently, ongoing research aims at improving the delivery and stability of RNAi agents, which is essential for advancing the clinical applicability of RNAi in cancer therapy [207]. For example, novel exosome-based delivery systems have been engineered to carry siRNAs or shRNAs that targeted mutant *KRAS^G12D^* in multiple mouse models of pancreatic cancer [208].

In summary, RNAi offers a precise and flexible approach for gene silencing, providing a strategy for overcoming treatment resistance and enhancing chemotherapy efficacy by targeting lncRNAs and RBPs highlighted herein. While challenges related to delivery and stability persist, advancements in RNAi technologies hold promise for translating this innovative approach into effective clinical therapies for PDAC patients.

### 4.2. Antisense Oligonucleotides to Target lncRNAs and RBPs

Antisense oligonucleotides (ASOs) represent a powerful therapeutic strategy for targeting lncRNAs and RBPs. These short, single-stranded nucleic acids are designed to bind to complementary RNA sequences, providing a means to modulate gene expression at the transcript level. Importantly, their precise mechanisms of action depend on the specific design and chemical modification of ASOs. A major mechanism by which ASOs exert their effects is through their interaction with ribonuclease H (RNase H). This endogenous enzyme cleaves the RNA strand in RNA-DNA hybrids, and ASOs designed to leverage this interaction therefore contain a central DNA region flanked by chemically modified nucleotides [207,209]. Because RNase H cleavage occurs in the nucleus and cytoplasm, ASOs might be the preferred agents to manipulate the expression of lncRNAs that reside in the nucleus [209,210]. Another clinically relevant mechanism of action involves the regulation of alternative splicing by ASOs [211]. By binding to splice sites or regulatory sequences, ASOs can modulate the splicing patterns of target genes. Similarly, ASOs can function as steric blockers to prevent intra- and intermolecular RNA-RNA as well as RNA-DNA and RNA–protein interactions. This mode of action could be harnessed to inhibit mRNA translation, regulate polyadenylation or interfere with miRNA-mediated RNAi. Hence, ASOs similar to RNAi agents offer high specificity and versatility, and their design can be tailored for particular applications, enhancing their therapeutic potential. Furthermore, ASOs generally have manageable toxicity profiles, as they can be engineered to avoid interactions with non-targeted RNAs and proteins. Nevertheless, ASO-based therapies also face similar limitations like other RNA-based agents, particularly regarding their specific delivery. ASOs are often susceptible to rapid degradation in the bloodstream, which can hinder their effectiveness. To reach target cells, ASOs may require specialized delivery systems, such as nanoparticle-based carriers or conjugation to cell-penetrating peptides [207,212]. Thus, advancements in delivery strategies are crucial for improving the therapeutic efficacy of ASOs and ensuring that they effectively reach their intended targets within cancer cells.

In conclusion, ASOs are powerful agents that enable the targeting of nuclear and cytoplasmic protein-coding and non-coding transcripts. By inhibiting the expression of critical PDAC-associated lncRNAs and RBPs, ASOs can potentially disrupt the pathways that support tumour growth and survival. However, innovative ASO delivery systems need to be developed to enhance the effectiveness of these therapies in the fight against pancreatic cancer.

### 4.3. Small-Molecule Inhibitors

In addition to the aforementioned oligonucleotide-based targeting strategies, small molecules comprise another interesting approach to target lncRNAs and RBPs. These molecules are either rationally designed to bind to specific regions within their targets or they can be discovered using unbiased screening approaches. The major mechanism of action of such small-molecule inhibitors is to interfere with intra- and/or intermolecular interactions, thereby, for example, causing RNA misfolding or blocking the interaction between RBPs and their target transcripts. Indeed, several small-molecule inhibitors have been developed in recent years, and interested readers are referred to excellent reviews on this exciting topic [17,213,214,215,216,217,218]. Importantly, small-molecule inhibitors offer several advantages in targeting lncRNAs and RBPs. For example, by designing small molecules that bind to specific domains or structural elements only present in the target lncRNA or RBP, these molecules can be engineered for specificity, allowing for targeted inhibition with potentially minimal impact on non-target RNAs or proteins. Furthermore, many small-molecule inhibitors are designed for oral bioavailability and are optimized for pharmacokinetics and pharmacodynamics, which can simplify their administration and improve patient compliance. Compared to oligonucleotides and viral delivery systems, small molecules are typically easier and cheaper to synthesize and modify. Nevertheless, the identification of specific and effective small-molecule inhibitors to target RNAs and RBPs is not a trivial task given the lack of enzymatic activities that would provide an easy read-out for measuring on-target activity. Moreover, the lack of suitable binding pockets within most RBPs as well as the conserved structure of commonly found RNA-binding domains and therefore the lack of RBP-specific structural motifs as a suitable target region to disrupt intermolecular interactions complicates the rational design of small molecules. Moreover, combinatorial recognition of target RNA sequences or structures may be a common phenomenon, which makes it difficult to design small molecules that are able to block multiple binding sites. In the case of lncRNAs, structural information is often not available, and functionally relevant regions within the transcript are often unknown. Last but not least, a small molecule binding to its lncRNA or RBP target might not be sufficient to interfere with the activity of the respective target. However, once identified, specific small molecule binders can be converted to degraders as will be discussed in the next paragraph.

### 4.4. Targeting of lncRNAs and RBPs via Proximity-Inducing Bifunctional Molecules

The targeted degradation of disease-relevant proteins is a therapeutic concept that has gained lots of attention in recent years and has been transferred recently to the RNA world as well. The general idea is to induce proximity between a target molecule, e.g., RBP or lncRNA, and a machinery that is able to degrade the target. In order to achieve targeted protein degradation (TPD), so-called proteolysis-targeting chimeras (PROTACs) have been developed. These are heterobifunctional small molecules consisting of two ligands: one that binds a specific target protein and another one that binds an E3 ubiquitin ligase. Both ligands are joined by a linker, and the simultaneous binding of both ligands to their intended proteins induces the ubiquitination and subsequent degradation of the target protein [219]. This concept was recently applied to degrade the splicing factor SF3B1 as well as the eukaryotic translation initiation factor EIF4E [220,221]. An interesting variation of this concept, called RNA-PROTAC, has been developed and applied to degrade two RBPs, namely LIN28A and RBFOX1 [222]. The RNA-PROTAC approach requires a chimeric molecule which consists of a short oligo-ribonucleotide corresponding to the consensus binding sequence of the RBP that is conjugated to a peptide derived from HIF1A, thereby enabling the recruitment of VHL, an E3 ubiquitin ligase. The adaptation of the concept of proximity-induced degradation to eliminate specific RNAs led to the development of ribonuclease-targeting chimeras (RIBOTACs). Here, small molecules that bind to the RNA of interest are modified to carry a short 2′-5′oligoadenylate or another small molecule that binds to and locally activates RNase L [223,224,225]. This approach was recently leveraged to convert strong yet inactive binding interactions into potent and specific modulators of RNA function [226]. Of note, additional ASO and aptamer-based strategies have been invented to engage RNase L in targeted RNA degradation [227,228,229,230]. Thus, the field is rapidly evolving, and the development and application of PROTACs and RIBOTACs to target RBPs and lncRNAs in pancreatic cancer might change the trajectories of this devastating disease.

In summary, several strategies enable the targeting of lncRNAs and RBPs, thereby opening new avenues for biomedical research and development. However, targeting these new classes of molecules comes with additional clinical challenges and limitations. For example, targeting RBPs with housekeeping functions, e.g., regulators of splicing or translation, might cause systemic on-target toxicities that need to be carefully assessed using appropriate preclinical models. In addition, off-target toxicities of the therapeutic molecules could also occur and should be taken into account. Furthermore, genes with redundant functions, e.g., members of a specific RBP family, might require concurrent targeting in order to achieve therapeutic effects and avoid treatment resistance. Hence, potential resistance mechanisms, which are hard to predict, should be investigated early on using in vitro and in vivo approaches including unbiased functional genetic screens and molecular studies in order to understand the mode of action and drug resistance. This could also enable the development of companion diagnostics to enhance safety and efficacy. Last but not least, it is unlikely that single-target/single-agent strategies will achieve an enduring and deep clinical response in cancer therapy. Therefore, targeting a specific RBP or lncRNA in combination with already approved treatment regimen, e.g., gemcitabine/nab-paclitaxel in the case of PDAC, might be a promising strategy that should be explored systematically.

## 5. Conclusions and Future Perspectives

LncRNAs and RBPs are critical regulators of chemoresistance, cancer stem cell properties and oncogenic signalling in PDAC, making them compelling candidates for therapeutic interventions and disease biomarkers. In particular, the oncofetal expression pattern of individual RBPs and the tissue- and cell-type specific expression of certain lncRNAs represent unique opportunities for making future therapies more specific and early detection more sensitive. Therefore, future research should focus, on the one hand, on elucidating the molecular functions of individual, disease-associated lncRNAs and RBPs as well as their interconnections to better understand the complex regulatory networks that drive PDAC progression. Importantly, interfering with these interactions could be a valid therapeutic strategy. For example, targeting *LINC01232* or *UCA1* might affect hnRNPA2/B1 expression and its oncogenic functions [93,102]. Alternatively, preventing the m6A-dependent interaction between *LINC00941* and IGF2BP2 could be a valid strategy to impair pancreatic cancer cell migration and invasion [231]. Additionally, *LINC00261* was shown to compete with the *MYC* mRNA for binding to IGF2BP1, suggesting that the overexpression of *LINC00261*, or maybe just parts of it, could be used to decrease oncogenic MYC protein expression in PDAC [232].

On the other hand, there is an urgent need for novel sensitive and specific biomarkers, since the only Food and Drug Administration (FDA)-approved blood biomarker CA 19-9 is limited in its applicability for surveillance due to its variability among different ethnic populations and its insufficient sensitivity and specificity [233,234]. Thus, prospective studies should explore several sources, e.g., blood, saliva, urine and exosomes, and analyze different molecular classes, as well as potentially combine multiple approaches, especially transcriptomics and proteomics, in order to evaluate lncRNAs and RBPs as early disease biomarkers for patient identification and stratification. In addition, high-risk populations, like patients diagnosed with chronic pancreatitis, should also be considered and thoroughly investigated in order to identify RBPs and lncRNAs that are associated with predisposing conditions [235,236].

With ongoing research and improvements in targeting and drug delivery, the inhibition of lncRNAs and RBPs holds great potential, and novel combinatorial approaches involving lncRNA- or RBP-targeting drugs as well as conventional (chemo)therapies or other targeted agents may enhance their therapeutic efficacy and pave the way for more effective treatments against one of the most challenging cancers.

## Figures and Tables

**Table 1:** Overview of common modes of action of long non-coding RNAs (lncRNAs).

Function	Mode of Action	Description	Examples
Enhancer action	cis/trans	A total of 30–60% of lncRNAs are transcribed from enhancer regions, and enhancer activity is modulated by the active transcription and splicing of enhancer lncRNAs [44,45,46]. These transcripts may potentiate enhancer activity by recruiting proteins that participate in the formation of chromatin loops between distal enhancers and their promoters or direct the recruitment of transcriptional activators to gene-proximal enhancers [47,48,49]. Similar mechanisms can also result in the repression of enhancer activity when lncRNA loci compete for enhancers with protein-coding genes [50].	*Hand2os1* [47,48] *CCAT1-L* [49] *PVT1* [50]
Chromatin architecture modulation	cis/trans	The process of X chromosome inactivation is dependent on the *cis*-acting lncRNA *XIST*, which spreads along and coats the inactive chromosome, ensuring dosage compensation [31,51]. Additionally, association with chromatin remodelling complexes is a common feature among lncRNAs [52]. The polycomp repressive complex 2 (PRC2) in particular is functionally dependent on RNAs and has been shown to interact with ~20% of human lncRNAs [52,53].	*HOTAIR* [30,54] *XIST* [31,51]
Biomolecular condensate formation	trans	Intracellular condensates are formed from RNA and protein interactions through liquid–liquid phase separation. Many of these compartments depend on architectural lncRNAs for their structures, compositions and functions [55].	*NORAD* [36] *NEAT1* [40] *MALAT1* [56]
miRNA sponge	trans	The target sites of miRNAs are found in mRNAs but are also present in many types of ncRNAs including lncRNAs. This has led to the hypothesis that all transcripts sharing binding sites for a particular miRNA may regulate each other by competing against each other [57]. An extensive number of publications have focused on the action of lncRNAs as competing endogenous RNAs (ceRNAs), but the hypothesis has also received criticism relating to the stoichiometries needed to support ceRNA regulatory networks [58].	*LINCMD1* [59], *PTENP1* [60,61]

**Table 2:** LncRNAs associated with KRAS/MAPK and TGF-β signalling in pancreatic cancer.

LncRNA	Expression in PDAC	Clinical Association	Function and Mechanism	References
*NUTF2P3-01*	upregulated	high expression correlates with tumour size; poor tumour differentiation; the tumour, node and metastasis (TNM) stage; lymphatic invasion; distant metastasis; and shorter overall survival (OS)	promotes proliferation, invasion, xenograft tumour growth and hepatic metastasis and acts as a ceRNA to sequester miR-3923 and increase KRAS expression	[90]
*MALAT1*	upregulated	high expression is associated with shorter OS	promotes proliferation, migration, invasion, and xenograft growth; reduces apoptosis; and acts as a ceRNA to sequester miR-217 and increase KRAS expression	[91]
*UCA1/CUDR*	upregulated	high expression correlates with an advanced T and N stage and is associated with poor OS	promotes proliferation, migration, drug resistance and xenograft tumour growth; reduces apoptosis; positively regulates MAPK/ERK and AKT/FAK signalling; enhances the interaction between KRAS and hnRNPA2/B1; and acts as a ceRNA to sequester miR-590-3p and increase KRAS expression	[93,95,96]
*ABHD11-AS1*	upregulated	expression correlates with poor OS and disease-free survival (DFS), an advanced TNM stage, increased distant metastasis and poor tumour differentiation	promotes proliferation and migration, reduces apoptosis and positively regulates PI3K/AKT signalling and EMT	[98]
*SNHG1*	upregulated	expression correlates with an advanced TNM stage and a larger tumour size	promotes proliferation, invasion and xenograft tumour growth; reduces apoptosis; and positively regulates PI3K/AKT signalling	[99]
*LUCAT1*	upregulated	increased expression linked to a larger tumour size and lymphatic invasion	promotes proliferation, invasion and xenograft tumour growth; reduces apoptosis; and positively regulates AKT and p38-MAPK signalling and the sponge of miR-539	[100,101]
*LINC01232*	upregulated	expression is positively correlated with nerve invasion, and high *LINC01232* levels are associated with worse outcomes	promotes migration and invasion and regulates the alternative splicing of ARAF by stabilizing hnRNPA2/B1, thereby activating MAPK/ERK signalling	[102]
*LINC00941*	upregulated	high expression is associated with a poor prognosis	promotes proliferation, is a downstream target of MAPK/ETS-1 signalling and increases E2F7	[104]
*PVT1*	upregulated	high expression is associated with an advanced clinical stage and lymph node metastasis (LNM)	promotes cell adhesion, viability, migration and invasion; enhances EMT via TGF-β/SMAD signalling; and downregulates SMAD4 and p21	[115,122]
*LINC00909*	upregulated	high expression is associated with poor OS and DFS and correlates with an advanced TNM stage, a larger tumour size, poor differentiation and LNM	promotes cell viability, colony formation, migration and xenograft tumour growth and metastasis; inhibits apoptosis; upregulates the expression of pluripotency factors; promotes the pancreatic cancer stem cell phenotype; activates the MAPK/JNK pathway; and destabilizes the *SMAD4* mRNA	[116]
*BC037916*	upregulated	increased expression correlates with a clinical stage and advanced T and N stages and is associated with poor OS	promotes proliferation, invasion and xenograft tumour growth; reduces apoptosis; positively regulates EMT via SMAD2/3 signalling; and upregulates JAK/STAT signalling	[117]
*LINC00462*	upregulated	expression is associated with a larger tumour size, poor tumour differentiation, a TNM stage and distant metastasis	promotes proliferation, migration, invasion, xenograft growth and invasion; reduces apoptosis and cellular adhesion; induces EMT through the activation of the TGF-β pathway via the upregulation of TGFBR1/2; and acts as a ceRNA to sequester miR-665	[118]
*LINC-PINT*	downregulated	Low plasma levels correlate with tumour recurrence and are associated with tumour size; a low *LINC-PINT* level in tumour tissues correlates with a poor prognosis after pancreatectomy	reduces cell proliferation and increases TGF-β1 expression in a pancreatic cancer cell line	[119,120]
*LINC00261*	downregulated	expression is subtype-dependent and shows an inverse correlation with tumour grade and stage, and high expression is associated with better OS	inhibits cell migration and invasion; TGF-β stimulation decreases *LINC00261* levels, and its genetic ablation leads to the downregulation of the E-cadherin mRNA and protein and the induction of an EMT programme	[121]

**Table 3:** RNA-binding proteins and their roles in pancreatic cancer.

RBP	Roles in PDAC	References
ELAVL1 (HuR)	upregulated in PDAC; associated with a poor prognosis; promotes proliferation, the cell cycle, migration and invasion; inhibits apoptosis; and might mediate gemcitabine sensitivity induced by promoting DCK expression; stabilized mRNAs in PDAC: *BARD1*, *DCK*, *DR5/TNFRSF10B*, *GPRC5A*, *IDH1*, *PRPS2*, *PDGFA*, *SNAI1*, *WEE1* and *YAP1*	[128,131,132,133,134,135,136,137,138,139,140,141,182]
IGF2BP1	upregulated in PDAC; associated with a poor prognosis; promotes proliferation, cell cycle progression, EMT, metastasis and gemcitabine resistance; is an m6A reader; and stabilized RNAs in PDAC: *SH3BP5-AS*, *E2F1* and *CDC25A*	[142,143,144,146,147,148,149,150,183]
IGF2BP2	upregulated in PDAC; associated with a poor prognosis; promotes proliferation and glucose metabolism; associated with an immune-suppressive microenvironment; is an m6A reader; and stabilized mRNAs in PDAC: *SLC2A1* and *CD274*	[142,143,154,155,156,157,158,159,183]
IGF2BP3	upregulated in PDAC; associated with a poor prognosis; frequently used as a biomarker (needle biopsy); promotes proliferation, adhesion, migration and invasion; associated with an immune suppressive microenvironment; is an m6A reader; and stabilized mRNAs in PDAC: *ARF6*, *ARHGEF4*, *CD44*, *SMS* and *UBE2K*	[142,143,157,161,162,164,165,166,167,168,169,170,171,172,173,174,183]
BICC1	upregulated in PDAC; expression associated with EMT and immune infiltration; and supports angiogenesis and chemoresistance by promoting LCN2 and CXCL1 expression	[176,177,178,179,180,181]
CELF2 (CUGBP2)	enhances stability but represses the translation of *VEGF* and *COX2* mRNAs and induced by curcumin	[184]
CSDE1 (UNR)	biomarker associated with a favourable prognosis and the immunogenic subtype	[185]
FUS	upregulated in PDAC, stabilizes the *NRF2* mRNA and suppresses oxidative stress and ferroptosis	[186]
hnRNPA2/B1	upregulated in early PDAC stages; inhibits apoptosis by suppressing *BCL-X(S)* splicing; regulates splicing of ARAF; and interacts with KRAS	[93,102,187,188]
hnRNPC	controls the mRNA stability of *IQGAP3* to promote cell proliferation, migration, invasion and metastasis	[189]
hnRNPF	part of a druggable super-enhancer network that promotes PDAC cell and tumour growth and stabilizes the *PRMT1* mRNA to promote *MYC* expression	[190]
hnRNPL	upregulated in PDAC, associated with a poor prognosis and promotes migration and EMT	[191]
LIN28B	upregulated in PDAC, associated with metastasis and a poor prognosis; promotes proliferation, stemness, migration and EMT; targets in PDAC (via de-regulating let-7): *MYC*, *HMGA2*, *KRAS*, *TET3*, *WNT5A* and *PCSK9*	[192,193,194]
MEX3A	upregulated in PDAC, expression correlates with disease progression in human and mice; promotes cell cycle and gemcitabine resistance and stabilized mRNAs in PDAC: *CDK6*	[195]
MSI2	upregulated in PDAC; associated with metastasis and a poor prognosis; promotes proliferation, metastasis and chemoresistance; transcriptionally suppressed by KLF4; and controls HIPPO signalling by suppressing SAV1 and MOB1 translation; mRNA targets in PDAC: *NUMB*, *MOB1*, *SAV1*	[196,197,198,199]
QKI	upregulated in PDAC tumour cells and fibroblasts and promotes proliferation, EMT and metastasis	[200]
RBFOX2	prevents PDAC progression and metastasis and controls the splicing of *ABI1* mRNA to inhibit migration	[201]
RBM10	downregulated in PDAC; the RBM10 mutation in advanced-stage tumours is associated with favourable survival; regulates the splicing of *hTERT*	[202,203]
STRBP	Binds to the *HSATII* RNA to prevent EMT and invasion and controls isoform switching of *CLSTN1*	[204]
ZFR	upregulated in PDAC and promotes proliferation, cell cycle progression and invasion	[205]
